# Neuroprotective Effect of Morin Hydrate against Attention-Deficit/Hyperactivity Disorder (ADHD) Induced by MSG and/or Protein Malnutrition in Rat Pups: Effect on Oxidative/Monoamines/Inflammatory Balance and Apoptosis

**DOI:** 10.3390/ph15081012

**Published:** 2022-08-17

**Authors:** Hoda A. Salem, Nehal Elsherbiny, Sharifa Alzahrani, Hanan M. Alshareef, Zakaria Y. Abd Elmageed, Sadeem M. Ajwah, Ahmed M. E. Hamdan, Yahia S. Abdou, Omneya O. Galal, Marwa K. A. El Azazy, Karema Abu-Elfotuh

**Affiliations:** 1 Department of Pharmacy Practice, Faculty of Pharmacy, University of Tabuk, Tabuk 71491, Saudi Arabia; 2 Department of Clinical Pharmacy, Faculty of Pharmacy, Al-Azhar University, Cairo 11884, Egypt; 3 Department of Pharmaceutical Chemistry, Faculty of Pharmacy, University of Tabuk, Tabuk 71491, Saudi Arabia; 4 Department of Biochemistry, Faculty of Pharmacy, Mansoura University, Mansoura 35516, Egypt; 5 Pharmacology Department, Faculty of Medicine, University of Tabuk, Tabuk 71491, Saudi Arabia; 6 Department of Pharmacology, Edward Via College of Osteopathic Medicine, University of Louisiana at Monroe, Monroe, LA 71203, USA; 7 PharmD Program, Faculty of Pharmacy, University of Tabuk, Tabuk 71491, Saudi Arabia; 8 Faculty of Medicine, Ain Shams University, Giza 11591, Egypt; 9 Department of Pharmacology and Toxicology, Faculty of Pharmacy, Ahram Canadian University (ACU), Cairo 12451, Egypt; 10 Department of Biochemistry and Nutrition, Faculty of Women for Arts, Science and Education, Ain Shams University, Cairo 11566, Egypt; 11 Department of Pharmacology and Toxicology, Faculty of Pharmacy, Al-Azhar University, Cairo 11884, Egypt

**Keywords:** monosodium glutamate, protein malnutrition, attention deficit hyperactivity disorder (ADHD), Nrf2/HO-1, NF-kB, TLR4, NLRP3

## Abstract

Monosodium glutamate (MSG) is one of the most widely used food additives. However, it has been linked to protein malnutrition (PM) and various forms of toxicities such as metabolic disorders and neurotoxic effects. The current study is the first to explore the association between MSG, PM, and induced brain injury similar to attention-deficit/hyperactivity disorder (ADHD). Moreover, we determined the underlying mechanistic protective pathways of morin hydrate (MH)―a natural flavonoid with reported multiple therapeutic properties. PM was induced by feeding animals with a low protein diet and confirmed by low serum albumin measurement. Subsequently, rat pups were randomized into seven groups of 10 rats each. Group I, III, and VI were normally fed (NF) and groups II, IV, V, and VII were PM fed. Group I served as normal control NF while Group II served as PM control animals. Group III received NF + 0.4 g/kg MSG, Group IV: PM + 0.4 g/kg MSG, Group V: PM + 60 mg/kg MH, Group VI: NF + 0.4 kg/g MSG + 60 mg/kg MH and Group VII: PM + 0.4 kg/kg MSG + 60 mg/kg MH. At the end of the experimental period, animals were subjected to behavioral and biochemical tests. Our results showed that treatment of rats with a combination of MSG + PM-fed exhibited inferior outcomes as evidenced by deteriorated effects on behavioral, neurochemical, and histopathological analyses when compared to rats who had received MSG or PM alone. Interestingly, MH improved animals’ behavior, increased brain monoamines, brain-derived neuroprotective factor (BDNF), antioxidant status and protein expression of Nrf2/HO-1. This also was accompanied by a significant decrease in brain MDA, inflammatory markers (NF-kB, TNF-α and IL1β), and suppression of TLR4/NLRP3/caspase-1 axis. Taken together, MSG and/or PM are associated with neuronal dysfunction. Our findings suggest MH as a potential neuroprotective agent against brain insults via targeting Nrf2/HO-1 and hindering TLR4/NLRP3 inflammasome signaling pathways.

## 1. Introduction

Maternal malnutrition in early life, such as protein malnutrition (PM), is one of the major non-genetic factors that affect the development of brain functions. Malnourished children show behavioral and cognitive dysfunction as well as a reduction in measures of intelligence and visuo-spatial processing capabilities, along with increased impulsivity, inattention, and attentional dysregulation [1]. Recently, monosodium glutamate (MSG), the sodium salt of non-essential amino acid-glutamic acid widely used as a food additive and taste enhancer, has been investigated in the context of malnutrition [2]. MSG is a member of group of chemicals known as excitotoxins that interfere with brain chemistry and cause alteration in neurotransmitters and oxidative stress imbalance [3]. According to the study performed by González-Burgos [4], glutamate has dual actions in the human body based on its plasma concentration. The neonatal exposure to glutamate exerts a neuroprotective effect arising from of glia cells proliferation and favoring the survival of neurons. However, if the brain is deluged with higher levels of free glutamate than needed, multiple disorders are developed including attention-deficit/hyperactivity disorder (ADHD). The exact mechanism underlying this disorder has not been fully studied, although several hypotheses are proposed.

Earlier studies suggest that nuclear factor erythroid-2 related factor 2 (Nrf2) is a key transcription factor and master regulator of cell response to oxidative stress and has a neuroprotective effect in neurological disorders [5]. Nrf2 activation also initiates the expression of different antioxidant enzymes and proteins such as heme oxygenase-1 (HO-1), an important mediator of the Nrf2 pathway, which promotes cell survival and protection [6]. Interestingly, dysregulation in HO-1 activity is associated with progression of neuro-inflammation, aging, and other neurodegenerative diseases. Furthermore, HO-1 induction in rats’ brain has shown neuroprotection against oxidative stress and neuro-inflammation [7].

Morin hydrate (MH) (3,5,7,21,40–penta hydroxyl flavone) is a natural bioflavonoid that is abundantly found in fruits, vegetables, green tea, and red wine [8]. It exhibits a very low toxicity level, and its chronic administration is well tolerated. Growing evidence suggests that MH has beneficial effects against various neurodegenerative diseases by acting as a reactive oxygen spices (ROS) scavenger, potent antioxidant, anti-inflammatory and cell-protective agent [9]. Its protective effect is contributed to its ability to attenuate cell apoptosis, ROS production as well as inhibits the increase of caspase-3 activity [10]. As previously reported, there is a potential link between malnutrition and behavior disturbance in children such as in ADHD where glutamate levels are affected and abnormally disturbed in the brain. Therefore, the current study was designed to investigate the mechanism underlying the effect of MSG on ADHD-induced brain injury alone and in combination with PM fed rats. The study also suggests MH as a promising neuroprotective agent in treatment of malnutrition and ADHD-induced behavior using a rat-model. Our study has numerous advantages over previous studies [1,4]. For instance, our study includes comparative histopathological features in different brain regions, it evaluates the behavioral study for the output effect of neuronal damage and neuroprotective effect, it used three different pathways for evaluating the neuroprotective effect; oxidative stress, inflammatory and apoptosis pathways, it studies the accumulative effect of both PM and chronic administration of MSG. So, our study investigated the effect of PM on the brain and the protective role of MH on PM using different behavioral, biochemical, and histopathological tests.

## 2. Results 

### 2.1. The Level of Serum Albumin in PM and/or MSG—Fed Rat Pups

**Figure 1:**
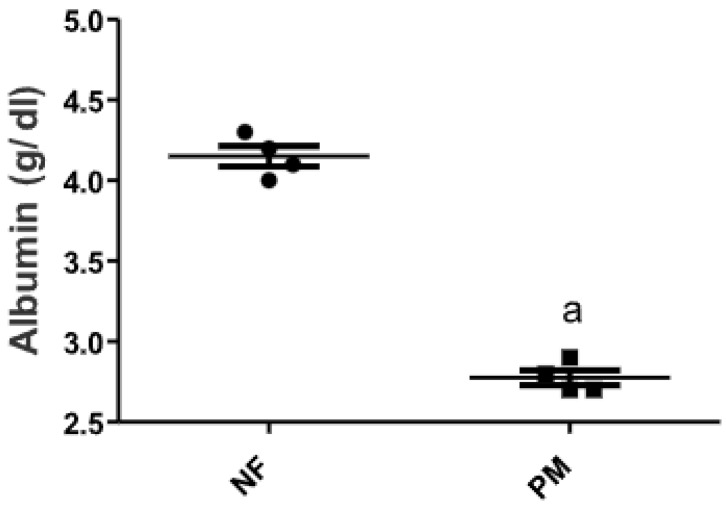
Serum albumin content of PM and/or MSG—fed rat pups. Serum albumin content was assessed in normally fed (NF) and protein malnourished (PM) rat pups. Data presented as mean ± SEM (*n* = 8). ^a^ depicts significant difference from control NF group at *p* < 0.05 using unpaired *t*-test. [The data of the effect of MH on the control NF is not shown as it is not significant].

Protein malnutrition (PM) induced by feeding rat pups with low protein diet (10% casein) for 3 weeks showed a marked 25% decrease in serum albumin from the corresponding control normal fed (NF) group (Figure 1).

### 2.2. Rat Behavior Tests

#### 2.2.1. MH Attenuates Alterations in Spatial Working Memory of PM and/or MSG—Fed Rat Pups

**Figure 2:**
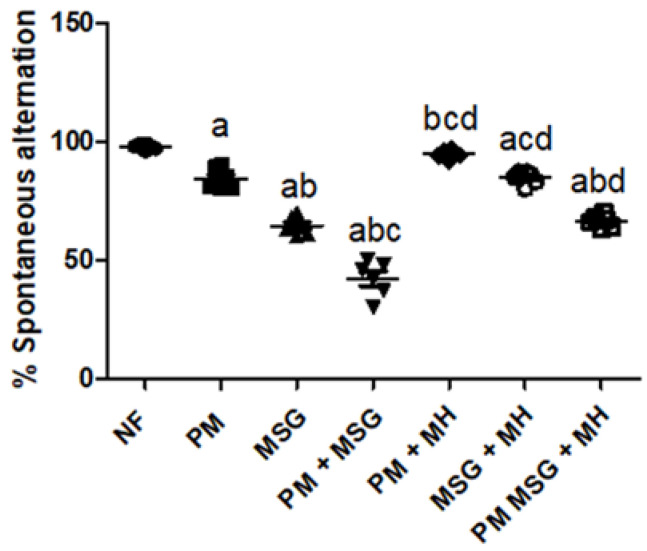
MH attenuates alterations in spatial working memory of PM and/or MSG—fed rat pups. Effect of 60 mg/kg Morin hydrate (MH) on low protein diet (PM) and/or 0.4 g/kg MSG on % spontaneous alteration in Y maze in rat pups. Data presented as mean ± SE (*n* = 6). a–d: Significant from NF, PM, MSG, or PM + MSG, respectively. Statistical analysis was performed using one-way ANOVA, followed by Tukey’s post hoc test (*p* < 0.05). [The data of the effect of MH on the control NF is not shown as it is not significant].

As shown in Figure 2, malnutrition induced by low protein diet (PM) for 11 weeks or by food-added 0.4 g/kg MSG for 8 weeks to NF pups showed a significant impaired working memory in animals depicted by a significant reduction in the percentage of spontaneous alteration in Y-maze by approximately 15% and 35%, respectively, relative to NF group. Addition of 0.4 g/kg MSG to PM fed animals showed the least spontaneous alteration by 55%. However, animals treated with 60 mg/kg MH significantly attenuated MSG-induced memory performance as seen by the increase in the percentage of alteration in all treated groups (MH + PM, MH + MSG, MH + PM + MSG) by 100%, 90% and 75%, respectively.

#### 2.2.2. MH Affects Swimming Ability in PM and/or MSG—Fed Rat Pups 

**Figure 3:**
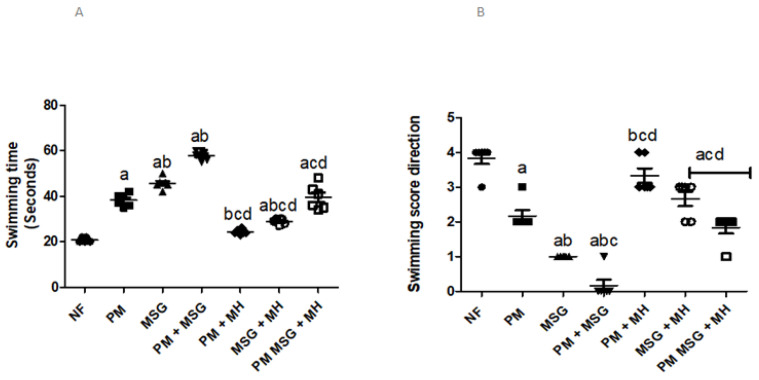
MH affects swimming test of PM and/or MSG—fed rat pups. Effect of 60 mg/kg Morin hydrate (MH) on low protein diet (PM) and/or 0.4 g/kg MSG on swimming time (**A**) and swimming direction score (**B**) using swimming test. Data presented as mean ± SEM (*n* = 6). a–d refers to significance from NF, PM, MSG, PM + MSG, respectively, at *p* < 0.05. Statistical analysis was performed using one-way ANOVA, followed by Tukey’s post hoc test. [The data of the effect of MH on the control NF is not shown as it is not significant].

As illustrated in Figure 3A,B, malnutrition induced by low protein diet (PM) for 11 weeks or by food-added 0.4 g/kg MSG for 8 weeks to NF pups showed that animals were associated with significant increase in cognitive dysfunction indicated by elongation in the swimming time (A) needed to climb the ramp by 100% and 125% respectively, and a worsening in direction score (B) by 50% and 75%, respectively. Food-added MSG to PM diet showed the worst behavior results among all induction groups. The combination of MSG + PM-fed was able to significantly increase swimming time by approximately 200% and decreased swimming direction score to almost 0 in comparison to NF animals. However, the addition of 60 mg/kg MH to all treatment groups (MH + PM, MH + MSG, MH + PM + MSG) improved the behavior outcome by decreasing swimming time and increasing swimming score in all tested animals.

#### 2.2.3. MH Restores Frequencies in OFT of PM and/or MSG—Fed Rat Pups 

**Figure 4:**
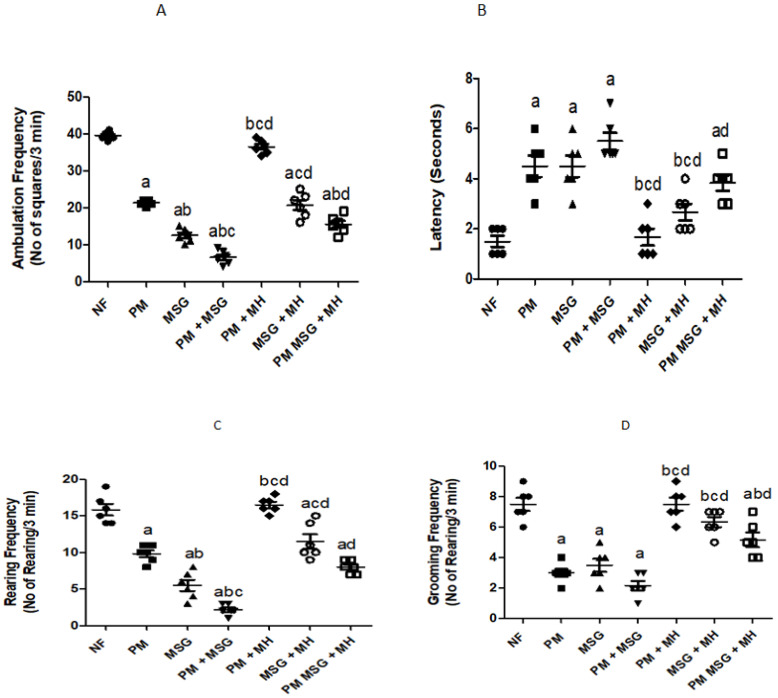
MH restores frequencies in OFT of PM and/or MSG—fed rat pups. Effect of MH (60 mg/kg) on low protein diet (PM) and/or 0.4 g/kg MSG on: ambulation (**A**); latency (**B**); rearing (**C**); and grooming (**D**) frequencies in OFT. Data presented as mean ± SEM (*n* = 6). a–d depicts significant difference from NF, PM, MSG, PM + MSG, respectively, at *p* < 0.05. Statistical analysis was performed using one-way ANOVA followed by Tukey’s post hoc test (*p* < 0.05). [The data of the effect of MH on the control NF is not shown as it is not significant].

Malnutrition induced by low protein diet (PM) for 11 weeks or by food-added 0.4 g/kg MSG for 8 weeks into NF pups was significantly reduced ambulation frequency (A) by 50% and 75%, respectively. Addition of 0.4 g/kg food-added MSG to PM diet decreased the ambulation frequency by 80% as shown in Figure 4A. The aforementioned observations were accompanied by non-significant increase in latency time (time required for animals to start moving) as PM diet or/and food-added 0.4 g/kg MSG showed sharp but non-significant increase in latency time by 150%, 150% and 200%, respectively, compared to NF group as presented in Figure 4B. In Figure 4C, PM diet or/and food-added 0.4 g/kg MSG significantly lowered rearing frequency by 33.3%, 67% and 83%, respectively, compared to NF control pups. In Figure 4D the results of grooming frequency of PM diet and/or 0.4 g/kg MSG surprisingly exhibited non-significant decrease by approximately 60%. However, animals treated with 60 mg/kg MH restored OFT frequencies as shown in Figure 4A–D.

### 2.3. MH Reestablishes Inflammatory Biomarkers in PM and/or MSG—Fed Rat Pups 

**Figure 5:**
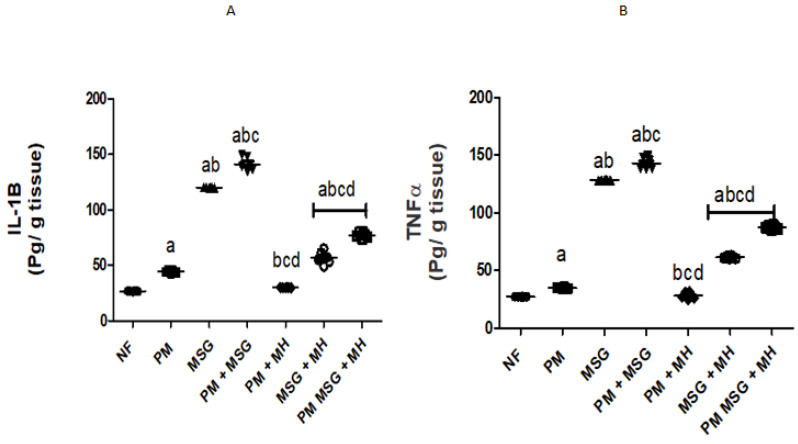

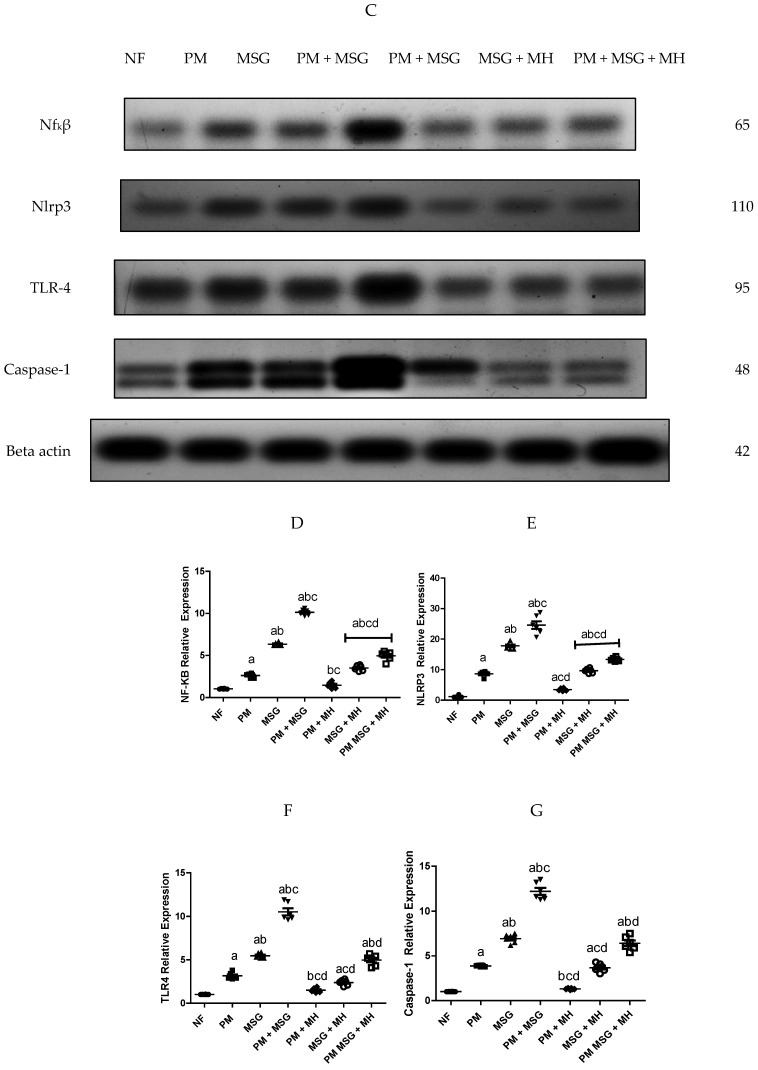
MH reestablishes inflammatory bio mediators of PM and/or MSG–fed rat pups: (**A**,**B**) effect of 60 mg/kg MH on brain inflammatory biomarkers of PM or/and 0.4 g/kg MSG rats measured by ELISA for IL-1β and TNF-α content; (**C**) immunoblotting represented nuclear fractions of NF-_K_B, NLRP3 inflammasome, TLR4 and capsase-1. β-actin was used as housekeeping protein to ensure equal protein loading; and (**D**–**G**) the intensities of protein expressions were quantified relative to β-actin and expressed as a fold change of NF-_K_B, NLRP3 inflammasome, TLR4 and caspase-1. Data presented as mean ± SEM (*n* = 6). a–d refers to significant difference relative to NF, PM, MSG, PM + MSG, respectively, at *p* < 0.05. Statistical analysis was performed using one-way ANOVA followed by Tukey’s post hoc test. [The data of the effect of MH on the control NF is not shown as it is not significant].

Malnutrition induced by low protein diet (PM) for 11 weeks or by food-added 0.4 g/kg MSG for 8 weeks into NF pups induced series of complexed inflammatory cascade and brain neurotoxicity mirrored by high IL-1β and TNF-α protein levels in rat serum as depicted in Figure 5A,B. PM fed pups showed a relative but significant increase in IL-1β and TNF-α levels by 25% and 20%, respectively while food-added 0.4 g/kg MSG exhibited significant massive release of both detected inflammatory biomarkers in comparison to NF animals. The two animal models showed an increase in protein expression of NF-κB, NLRP3, TLR4 I and caspase-1 activity in rats’ brains when compared with NF group as confirmed by Western blotting analysis (Figure 5C). The addition of 0.4 g/kg MSG to PM fed animals elevated the detected biomarkers as compared with NF and with each disorder alone. Animals treated with 60 mg/kg MH significantly declined the inflammatory biomarkers in brain tissue as compared to their untreated corresponding groups (Figure 5D–G). 

### 2.4. MH Balances Monoamines Neurotransmittors of PM and/or MSG—Fed Rat Pups

**Figure 6:**
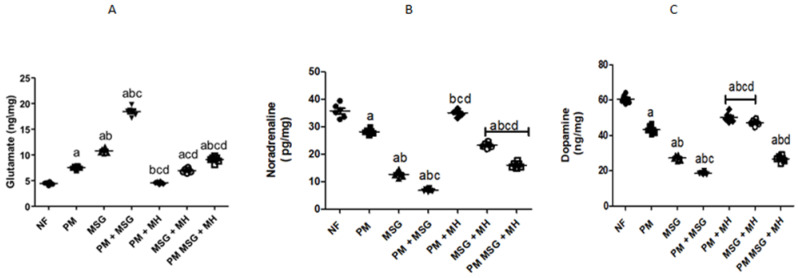

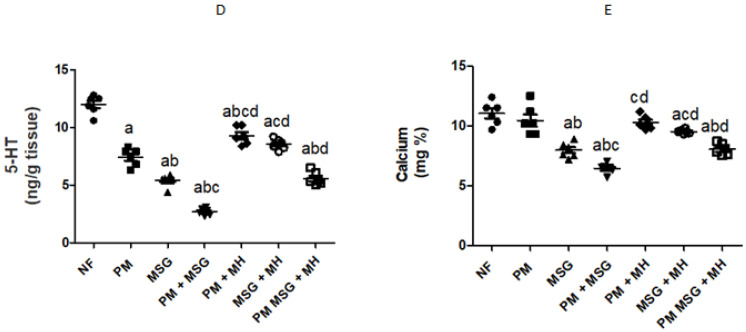
MH balances monoamines neurotransmitters of PM and/or MSG—fed rat pups. Effect of MH (60 mg/kg) on brain inflammatory biomarkers contents: (**A**) glutamate; (**B**) dopamine; (**C**) noradrenalin; (**D**) 5-HT; and (**E**) calcium levels using ELISA technique on low protein diet and/or 0.4 g/kg MSG. Data presented as mean ± SEM (*n* = 6). a–d depicts significant difference regarding NF, PM, MSG, PM + MSG groups, respectively, at *p* < 0.05. Statistical analysis was performed using one-way ANOVA followed by Tukey’s post hoc test. [The data of the effect of MH on the control NF is not shown as it is not significant].

As presented in Figure 6A–D, malnutrition induced by low protein diet (PM) for 11 weeks or by food-added 0.4 g/kg MSG for 8 weeks into NF pups showed Glutamate content was significantly increased in comparison to NF animals., While brain monoamines of dopamine, noradrenaline, and serotonin (5-HT) concentrations significant decrease, while In Figure 6E, low protein diet fed animals did not show any significance on brain calcium concentration, while food added 0.4 g/g MSG to NF animals decreased calcium concentration by 20%. The addition of 0.4 g/kg MSG to PM fed animals further decreased the contents of brain monoamines of dopamine, noradrenaline, 5-HT, and calcium as shown in Figure 6B–E. On the other hand, the combination of 0.4 g/kg MSG and PM was significantly increased glutamate concentration by approximately 15% in comparison to NF and as compared with each induced group alone (Figure 6A). MH (60 mg/kg) treatment of PM, food-added MSG, and PM + MSG increased the brain monoamines contents of dopamine, noradrenaline, 5-HT and calcium while it decreased glutamate concentration (*p* < 0.05) as compared to their corresponding PM, MSG and PM + MSG groups, respectively.

### 2.5. MH Modify Brain Oxidative Stress Biomarkers of PM and/or MSG—Fed Rat Pups

**Figure 7:**
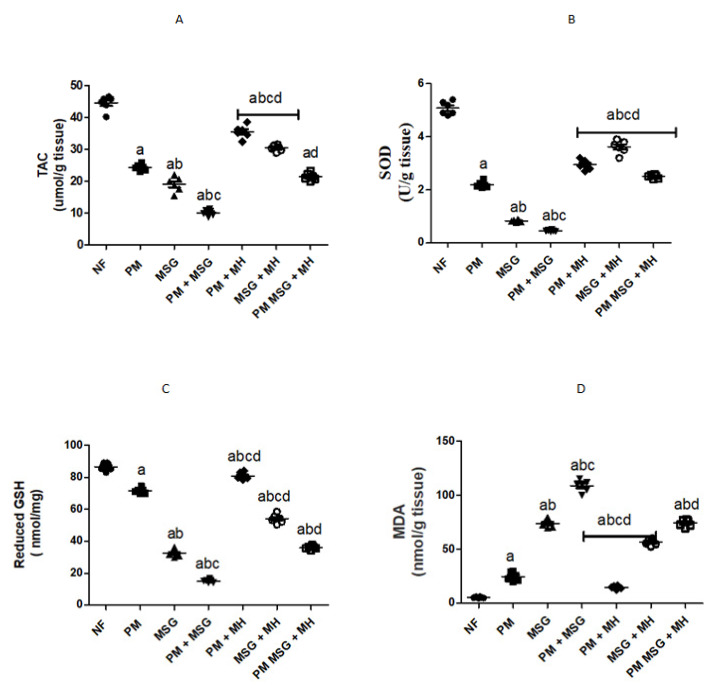

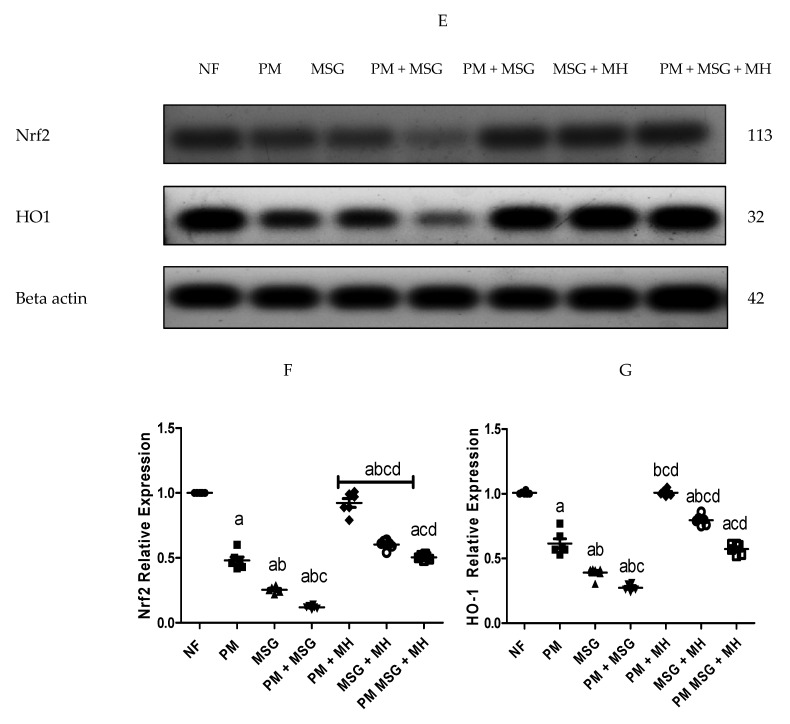
MH modify brain oxidative stress biomarkers of PM and/or MSG—fed rat pups. Effect of MH (60 mg/kg) (MH) on brain oxidative stress biomarkers of PM or/and MSG rats on the level of: TAC (**A**); SOD (**B**); reduced glutathione (**C**); and MDA (**D**) on the protein level using ELISA. Immunoblotting membranes represented nuclear fractions of Nf2 expression and HO-1 and β-actin was used a loading protein control (**E**). Relative protein expression of Nrf2 (**F**) and HO-1 (**G**). Data presented as mean ± SEM (*n* = 6). a–d depict significant difference regarding NF, PM, MSG, PM + MSG groups, respectively at *p* < 0.05. Statistical analysis was performed using one-way ANOVA, followed by Tukey’s post hoc test. [The data of the effect of MH on the control NF is not shown as it is not significant].

As shown in Figure 7, malnutrition induced by low protein diet (PM) for 11 weeks or by food-added 0.4 g/kg MSG for 8 weeks in rat pups significantly disrupted the oxidative stress balance in rats’ brain which was evidenced by colorimetric assessment of TAC (total antioxidant activity), SOD (superoxide dismutase), reduced glutathione (GSH) and MDA (malondialdehyde) content (Figure 7A–D). Moreover, both stressors decreased protein expression of Nrf2 and HO-1 in brain tissues as shown by Western blotting and protein quantification of Nrf2 and HO-1 (Figure 7E–G). The addition of 0.4 g/kg MSG to PM fed animals exhibited significant progressive imbalance between pro/antioxidant stress brain markers as compared with NF animals and with each stressor alone. Animals treated with 60 mg/kg MH was able to restore pro/antioxidant balance in rats’ brain by significantly reducing MDA and GSH contents and increase SOD and TAC levels as well as protein expression of Nrf2 and HO-1 in comparison with untreated groups. 

### 2.6. MH Adjust BDNF Content of PM and/or MSG—Fed Rat Pups 

**Figure 8:**
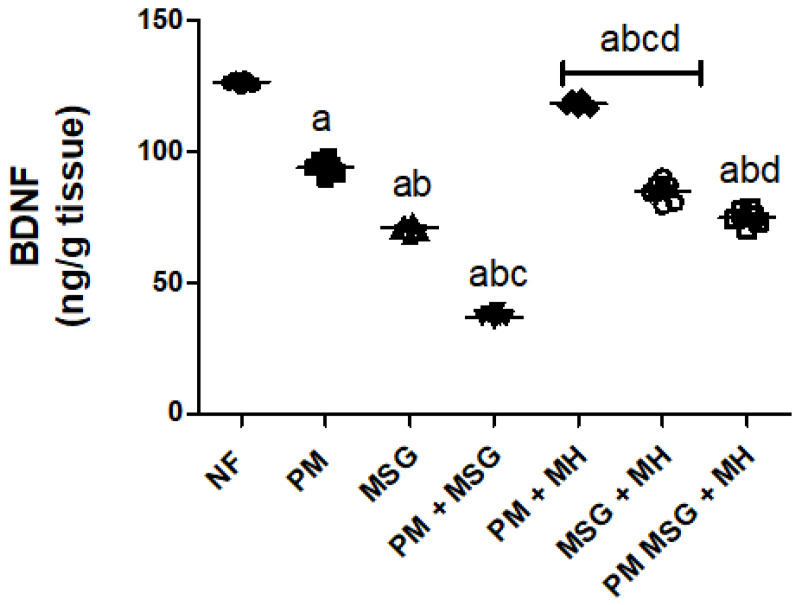
MH adjust BDNF content of PM and/or MSG—fed rat pups. Effect of 60 mg/kg MH on brain BDNF content of PM diet and/or 0.4 g/kg MSG. Data presented as mean ± SEM (*n* = 6). a–d refers to significant difference relative to NF, PM, MSG, PM + MSG groups, respectively, at *p* < 0.05. Statistical analysis was performed using one-way ANOVA, followed by Tukey’s post hoc test. [The data of the effect of MH on the control NF is not shown as it is not significant].

As shown in Figure 8, malnutrition induced by low protein diet (PM) for 11 weeks or by food-added 0.4 g/kg MSG for 8 weeks into NF pups significantly lowered BDNF content in brain homogenate as revealed by ELISA analysis. Additionally, the administration of 0.4 g/kg MSG to PM fed animals showed further significant decrease in BDNF content as compared to NF animals and to each induced group alone. Animals treated with 60 mg/kg MH of PM and/or 0.4 g/kg MSG increased the content of BDNF (*p* < 0.05) as compared to their untreated corresponding groups. 

### 2.7. MH Correct GFAP Brain Content of PM and/or MSG—Fed Rat Pups 

**Figure 9:**
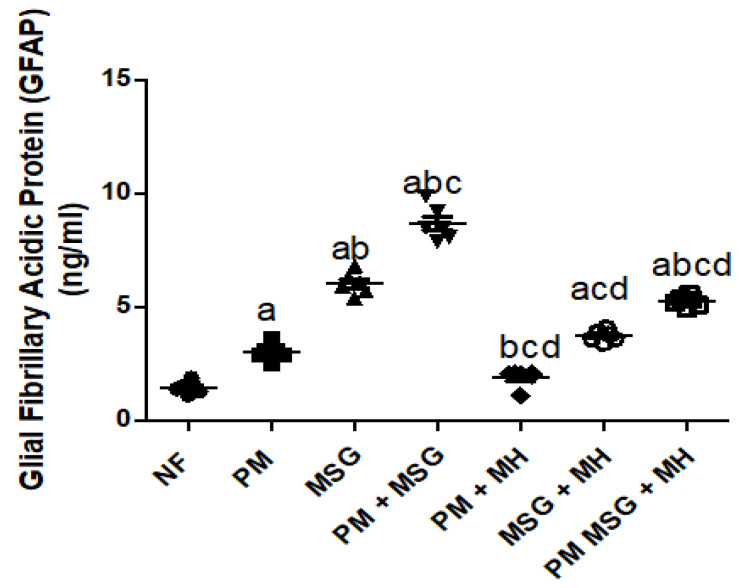
MH correct GFAP brain content of PM and/or MSG—fed rat pups. Effect of 60 mg/kg MH on brain GFAP content of PM diet and/or 0.4 g/kg MSG. Data presented as mean ± SEM (*n* = 6). a–d represents significant difference to NF, PM, MSG, PM + MSG groups, respectively, at *p* < 0.05. Statistical analysis was performed using one-way ANOVA, followed by Tukey’s post hoc test. [The data of the effect of MH on the control NF is not shown as it is not significant].

As presented in Figure 9, malnutrition induced by low protein diet (PM) for 11 weeks or by food-added 0.4 g/kg MSG for 8 weeks into NF pups significantly elevated Glial Fibrillary Acidic Protein (GFAB) content in brain by 50% and 200%, respectively, in brain homogenate as revealed by ELISA results. The addition of 0.4 g/kg MSG to PM fed animals further increased GFAP to approximately 300% (*p* < 0.001). The addition of 60 mg/kg Morin hydrate (MH) to PM fed animals brought GFAP back close to the normal level as compared to NF fed pups. MH was able as well to significantly decrease GFAP content when added to 0.4 g/kg MSG and 0.4 g/kg MSG + PM but to a lesser extent by approximately 33% and 30%, respectively.

### 2.8. MH Reverts Apoptotic/Anti-Apoptotic Markers of PM and/or MSG—Fed Rat Pups

**Figure 10:**
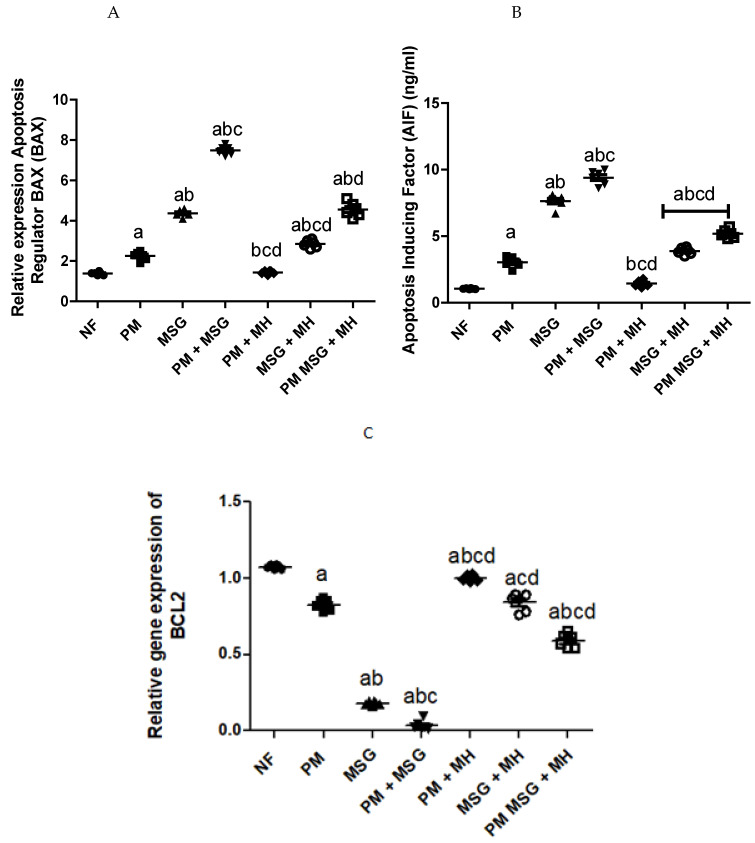
MH improve apoptotic/anti-apoptotic markers of PM and/or MSG—fed rat pups. Effect of 60 mg/kg MH combined with PM diet and/or 0.4 g/kg MSG on brain activity of: *Bax* (**A**); AIF (**B**); and *Bcl-2* (**C**) as apoptosis related markers in rats. Data presented as mean ± SEM (*n* = 6). a–d depicts significant difference compared to NF, PM, MSG, PM + MSG groups, respectively, at *p* < 0.05. Statistical analysis was performed using one-way ANOVA, followed by Tukey’s post hoc test. [The data of the effect of MH on the control NF is not shown as it is not significant].

A signified pro-apoptotic impact on neuronal cells was aberrant in animals exposed to malnutrition induced by low protein diet (PM) for 11 weeks or by food-added 0.4 g/kg MSG for 8 weeks into NF pups (Figure 10). This effect was reflected by upregulation of apoptotic regulator *Bax* and an increase in apoptosis inducing factor (AIF; pro-apoptotic), whereas *Bcl-2* (anti-apoptotic) was downregulated. The addition of 0.4 g/kg MSG to PM diet has severely affected *Bcl-2* levels in brain tissues and decreased its activity to almost zero. The AIF contents of brain homogenate was revealed by ELISA while *Bax* and *Bcl-2* were measured by qPCR. Interestingly, the addition of 60 mg/kg MH revealed a significant anti-apoptotic capability by reverting the post concussive imbalance in apoptotic markers encountered after malnutrition. 

Quantitative analysis of the Bax and Bcl-2 mRNA expression was performed and the Bax/Bcl-2 ratios are listed in Table 1. Interestingly, we found that a Bax/Bcl-2 ratio >1 and was statistically significant (*p* < 0.05) (Figure 10). 

### 2.9. MH Alter Histopathological Brain Specimen of PM and/or MSG—Fed Rat Pups

**Figure 11:**
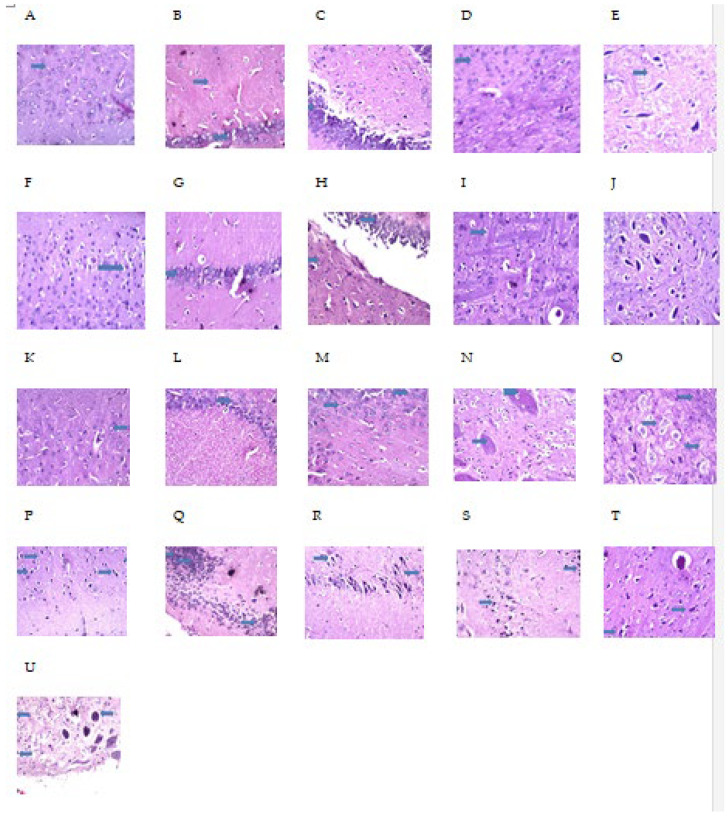
Photomicrographs (**A**–**E**): Transverse brain tissue sections from **control NF** group illustrating no histopathological alteration in: cerebral cortex (**A**); hippocampus (**B**); striatum (**C**); and substantia nigra (**D**,**E**) (arrows). Photomicrographs (**F**–**J**): transverse brain tissue sections from control **PM animals’** cerebral cortex showed focal nuclear pyknosis and degeneration in the neuronal cells (**F**). There was no histopathological alteration in the hippocampus as well as in the striatum (**G**–**I**) (arrows). Atrophy was detected in some neurons of the substantia nigra (**J**). Photomicrograph (**K**–**O**): Transverse brain tissue sections from **MSG-treated** animals illustrates no histopathological alteration in cerebral cortex (**K**) and hippocampus (**L**,**M**) (arrows). Focal plagues formation as well as intracellular neuronal oedema were detected in striatum (**N**). Intracellular oedema was also observed in the neurons of substantia nigra (**O**) (arrows). Photomicrographs (**P**–**U**): Transverse brain tissue sections from **MSG-treated PM animals** show that nuclear necrosis and degeneration were observed in the neurons of cerebral cortex (**P**) (arrows), associated with focal gliosis (**Q**) (arrows). The pyramidal cells of the hippocampus as well as the neurons of the fascia dentate, striatum and substantia nigra showed nuclear pyknosis and degeneration with congestion in the blood vessels (**R**–**U**) (arrows). Magnification was 40×.

**Figure 12:**
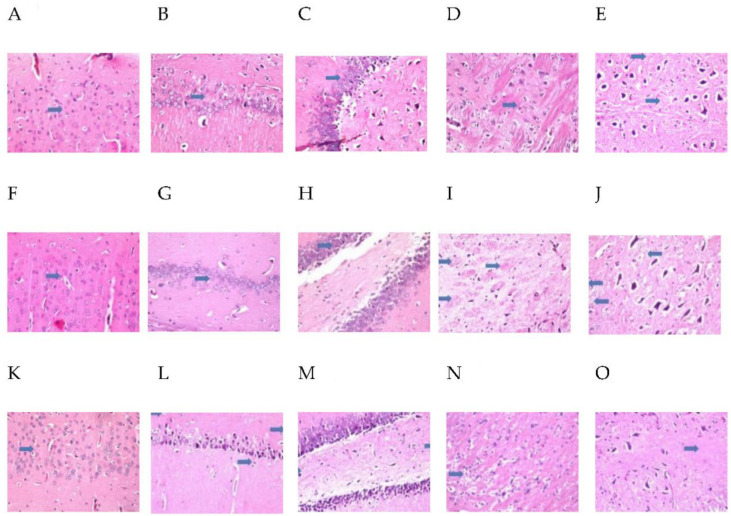
Photomicrographs (**A**–**E**): transverse brain tissue sections from **MH treated-PM** animals show no histopathological alteration in cerebral cortex (**A**) (arrows), hippocampus (subiculum, fascia dentate and hilus) (**B**,**C**) (arrows), striatum and substantia nigra (**D**,**E**) (arrows). Photomicrographs (**F**–**J**): Transverse brain tissue sections from **MH + MSG of NF** animals illustrate normal histological structure of the cerebral cortex and hippocampus (subiculum, fascia dentate and hilus) (**F**–**H**) (arrows). Focal fine plagues were detected in striatum (**I**) (arrows) and there was atrophy seen in some neuronal cells in the substantia nigra (**J**). Photomicrographs (**K**–**O**): Transverse brain tissue sections from **MH + MSG in PM fed** animals display no histopathological alteration in the cerebral cortex (**K**) (arrows). Nuclear pyknosis and degeneration were observed in some neuronal cells of the subiculum as well as the fascia dentate in the hippocampus (**L**,**M**) (arrows). The striatum showed intracellular oedema in the neuronal cells (**N**) (arrows). Mild atrophy was detected in the cells of substantia nigra (**O**) (arrows). Magnification was 40×.

The effect of MH treatment on PM and/or MSG—fed animals in brain are shown in Figure 11 and Figure 12. Control NF group illustrated no histopathological alterations, while control PM animals’ cerebral cortex showed focal nuclear pyknosis and degeneration in the neuronal cells with no alteration in the hippocampus as well as in the striatum and atrophy was detected in some neurons of the substantia nigra. Sections from MSG-treated animals showed no histopathological alteration in cerebral cortex and hippocampus. However, focal plagues formation as well as intracellular neuronal oedema were detected in striatum. Sections from MSG-treated PM animals showed that nuclear necrosis and degeneration in the neurons of cerebral cortex, associated with focal gliosis. The pyramidal cells of the hippocampus as well as the neurons of the fascia dentate, striatum and substantia nigra showed nuclear pyknosis and degeneration with congestion in the blood vessels.

MH treated-PM animals showed no histopathological alteration in cerebral cortex, hippocampus, striatum, and substantia nigra. MH + MSG of NF animals illustrated normal histological structure of the cerebral cortex and hippocampus. Focal fine plagues were detected in striatum and there was atrophy seen in some neuronal cells in the substantia nigra. Sections from MH + MSG in PM fed animals displayed no histopathological alteration in the cerebral cortex. Nuclear pyknosis and degeneration were observed in some neuronal cells of the subiculum as well as the fascia dentate in the hippocampus. The striatum showed intracellular oedema in the neuronal cells. Mild atrophy was detected in the cells of substantia nigra. 

## 3. Discussion

Earlier studies have correlated MSG to malnutrition and behavior disturbance [11], but the pathological mechanism mediating this effect is poorly understood. Therefore, to our knowledge, the current study is the first to explore the association between MSG, malnutrition and ADHD behavior disturbance in attempt to determine the nutritive importance in such disorder alongside the possible mechanistic pathway of MH as neuroprotective agent. 

In the current study, rats’ cognitive functions were affected after the administration of MSG and showed decline in their short-term memory as observed from their performance in Y-maze test. This finding was supported by previous studies [12,13]. However, the cognitive affection was much higher in PM-fed rats receiving MSG compared to normal counterparts. Since the hippocampus in the forebrain plays a major role in controlling memory and regulating learning functions and thinking [14], the decline shown in Y-maze test could be attributed to MSG neurotoxic effect on forebrain neurons including the hippocampus. Additionally, other studies reported such decline as a result of alteration in brain neurotransmitters [15]. Thus, the current study evaluated the effect of PM and/or MSG fed pups on different brain neurotransmitters such as glutamate, NA, 5-HT, dopamine, and calcium content in hippocampus. PM-fed animals showed significant high levels of glutamate and decrease in dopamine, noradrenaline, and serotonin in comparison to NF rats. The administration of MSG to PM rats reported the highest elevation of glutamate and lowest levels of dopamine, NA and 5-HT compared to MSG-fed animals only. Serum 5-HT levels are linked to memory and learning impairments in animals as also confirmed in our results [16]. In the context of our findings, the depletion of 5-HT stores in mice increases MSG-induced lethality [17]. In addition, high glutamate levels were reported to decrease 5-HT synthesis and increase its metabolism [18]. Moreover, subcutaneous injection of MSG in newborn rats resulted in high 5-HT uptake in cerebral cortices [19]. Interestingly, PM-fed animals showed no changes in brain calcium levels compared to NF animals. On the other hand, the administration of MSG to NF-fed pups showed a little but significant decrease in brain calcium levels and the addition of MSG to PM fed-animals showed a further decline in calcium level [20]. Demonstrated that glutamate levels affect intracellular Ca^+2^ oscillation frequency, leading to an increase in the content of intracellular calcium. Increased calcium influx affects the release of other neurotransmitters as well as activates cell death by releasing proapoptotic factors and generation of ROS [21]. The outcome of calcium evaluation in the present study strongly supports our finding that ADHD is associated with impaired Ca^+2^ hemostasis. Further behavior investigation of PM and/or MSG-fed animals reported low swimming score and changes in locomotor activity by swimming test and OFT. These behavioral findings may be attributed to depletion of brain monoamines such as glutamine and acetylcholine beside the nuclear pyknosis and degeneration detected in the cerebral cortex and hippocampus of PM, MSG, and PM + MSG rats’ brains as evidenced by biochemical and histopathological results. Further, changes in open field activities, anxiety-related and stimulation of the brain reward system are attributed to the imbalance between brain glutamate/glutamine levels after MSG ingestion [22].

The exposure of pups to PM and/or MSG showed a marked disturbance in pro/anti oxidative stress markers and inflammatory mediators. This was revealed by a significant decrease in TAC, SOD, reduced GSH and significant increase in pro-oxidant content of MDA along with a decrease in brain defense mechanism of Nrf2 and HO-1 in comparison with NF rats. Moreover, they revealed extensive significant upregulation of caspase-1, NF-κB, TLR4 and NLRP3 expression with a marked increase in brain proinflammatory TNF-α and IL-1β. Accordingly, this explains the pathological downregulation mechanism of BDNF contents which favor cell protection and survival. Our findings come in accordance with several previous studies investigated the oxidative insults of MSG and the generation of free radicals [23,24]. SOD is considered the first line of defense against released oxygen (O_2_^−^) radicals. The observed decrease in SOD activity could explain the failure of the antioxidant system to overcome the released free radicals [25]. The significant inhibition of SOD activity in the current study verifies the oxidative stress status induced by PM and/or MSG. Further, histological examination showed focal plagues as well as intracellular neuronal oedema in brain striatum in MSG-treated animals. Brain tissue sections from MSG-treated PM animals show nuclear necrosis and degeneration in the neurons of cerebral cortex with congestion in hippocampal blood vessels. 

In the current study, brain proinflammatory cytokines, IL-1β and TNF-α, showed a significant increase and overexpression of NLRP3, NF_k_B and TLR-4 proteins. Oxidative stress leads to generation of free radical metabolites with consequent imbalance and disturbance of the endogenous antioxidant defense mechanism which in turn activates pro-inflammatory cytokines and contribute to ADHD. Interestingly, Nrf2 activity and expression has shown an important role in brain neurogenesis and neuroprotection. Nrf2 exerts both antioxidant and anti-inflammatory effects as the upregulation of Nrf2 transcription genes blocks proinflammatory transcription factor NF-κB which is activated by oxidative stress with subsequent decrease in transcription of pro-inflammatory cytokines such as IL-1β and TNF-α [26]. Focusing on role of inflammasome, especially in brain inflammation, NF-κB/TLR4/NLRP3 inflammasome initiate signaling pathway. Initially, NF-κB is activated via the TLR pathway which in turn triggering the production of both NLRP3 inflammasome as well as IL-1β and IL-6. The activation of NF-κB through TLR pathways may induce a synergistic increase in pro-inflammatory factors which in turn activates cell apoptosis [27]. 

As such, the results of the current study demonstrate that PM and/or MSG-treated animals resulted in triggering apoptosis in brain tissue as evident by downregulation of anti-apoptotic marker *Bcl-2* and an increase in AIF levels and upregulation of *Bax* compared to NF pups. 

GFAP is an important cytoskeletal component of astrocytes and closely associated with brain homeostasis as well as maintenance of brain functions. Though, astrocytes play an important role in neurogenesis [28], they initiate inflammatory events and generate inflammatory cytokines when excessively activated. Herein, PM and/or MSG-treated animals showed a significant increase in GFAP content as compared to NF group. 

MH has revealed its efficiency in guarding against behavioral and neurological exacerbations as well as significantly abate all undesirable effects associated with brain insults resulting from malnutrition (PM) and MSG ingestion in rat pups. It is recognizable that MH was able to correct behavior deviations after preventing spatial memory deficits, learning disabilities and immobility as observed in Y-maze, OFT and swimming test. Notably, MSG to already malnourished animals with exacerbated brain injury showed significant behavioral correction by MH treatment. Additionally, MH showed a better improvement in malnourished rats in comparison to MSG-treated group. This was further proved by histopathological examination, where MH treated-PM animals demonstrated no histopathological alterations in hippocampus and cerebral cortex. In addition, sections from MH treated with MSG of NF animals exerted normal histological structure of the cerebral cortex and hippocampus. However, some atrophy was observed in some neuronal cells in the substantia nigra. MH treatment in MSG-PM fed animals displayed no histopathological alterations in the cerebral cortex but nuclear pyknosis and degeneration was observed in some neuronal cells of the subiculum as well as the fascia dentate in the hippocampus. Moreover, the striatum showed intracellular oedema in the neuronal cells and atrophy was also detected in the cells of substantia nigra. 

The biochemical findings in the present study prove the prophylactic effect of MH against PM and/or MSG. MH was able to bring the measured brain indices within the normal range as corroborated by: (i) decrease neuroinflammation by dramatically suppress the expression of NF-κB, NLRP3, TLR4, caspase1 protein and decrease NF-κB-mediated cytokines, namely TNF-α and IL-1β. (ii) enhance animals’ brain monoamines of 5HT, dopamine, calcium and NE with decrease in glutamate levels which may explain the largely preserved integrity of hippocampal neurons detected in MH-treated rats with enhanced behavior, motor and retrieval of spatial memories. (iii) ameliorated oxidative stress biomarker displayed by upregulation of Nrf-2/HO-1 expression with increased of SOD, TAC and reduced GSH contents along with decreased in MDA level. (iv) evoked neurogenesis indicated by BDNF improvement. (iv) decreased neuroinflammation reported by the suppression of protein expression of NF-kB/TLR4/NLRP3 pathway as it down streamed effectors, namely: TNF-α, IL-1β content besides. (v) decrease of procaspase-1 protein expression. (vi) limit neuronal damage by decreasing GFAP expression levels, finally. (vii) revert the dysregulated apoptotic/anti-apoptotic markers encountered after malnutrition and/or the exposure to MSG. 

MH has notably proved to possess potent free radical scavenging ability and potent hepatoprotective effects [29,30,31]. Several studies have demonstrated that the administration of MH significantly prevented liver injury, inflammation, and fibrosis by enhancing Nrf2 regulated survival mechanism [32,33,34,35]. Additionally, MH has been reported to reduce hepatic inflammation-associated lipid accumulation in high fructose-fed rats via inhibition of sphingosine kinase 1/sphingosine 1-phosphate signaling pathway [36,37]. Another recent study established that MH could protect LPS/d-GalN-induced acute liver injury by activating Nrf2 signal pathways and inhibiting NF-κB activation [35]. Concerning the effect MH on memory impairment and hippocampal neurodegeneration, MH exerted a neuroprotective effect against chronic unpredictable stress-induced memory impairment and cortical neurodegeneration in animals via inhibition of oxidative/nitrergic stress, release of pro-inflammatory cytokines, and downregulation of iNOS and NF-_k_B. These findings offer an additive view for exploiting MH as an alternative neuroprotectant agent against stress-induced memory dysfunction [38]. It is suggested that MH has anti-atherosclerotic beside its anti-inflammatory effect by inducing autophagy through stimulation of cAMP/PKA/AMPK/SIRT1 signaling pathway [39].

## 4. Materials and Methods 

### 4.1. Drugs and Chemicals

**Figure 13:**
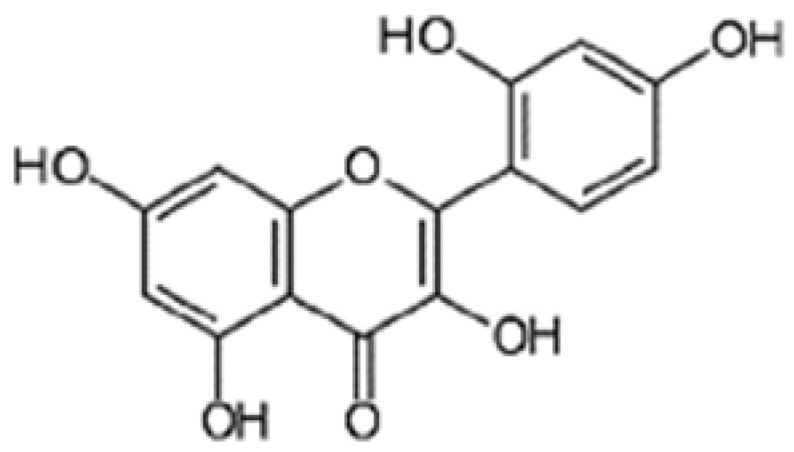
Chemical structure of Morin Hydrate.

Monosodium glutamate (MSG) and Morin hydrate (MH) (Figure 13) were purchased from Sigma-Aldrich Co. (St. Louis, MO, USA). MSG and MH were daily freshly prepared in doubled distilled water and given via intragastric tube at a dose of 0.4 g/kg and 60 mg/kg body weight according to [40,41], respectively. The protocol was approved by the Ethics Committee for Animal Experimentation at the Faculty of Pharmacy, Al-Azhar University, Cairo, Egypt (Protocol # 319/2022). The standard rodent protein diet was supplied from El-Nasr Company, Abu Zaabal, Cairo, Egypt. 

### 4.2. Animals

Seventy healthy weaning Sprague-Dawley male rat pups, weighing between 25 ± 5 g were included in this study. The pups were obtained from the Nile Pharmaceuticals Company, Cairo, Egypt. The animals were acclimatized at the animal facility for one week preceding the inception of the experiment. They were housed in standard stainless-steel cages and kept under constant experimental conditions (25 ± 2 °C, relative humidity 55 ± 10%, and 12-h light-dark cycle). The rats were fed a normal pellet diet and water ad libitum. The pups were handled according to the recommendation in the Guideline for the Care and Use of Laboratory Animals of the National Institute of Health (NIH Publication No. 8023, revised 1978). 

### 4.3. Diet

All animals were fed standard protein (NF) rodent chow during the first week of acclimatization. The standard protein diet contains casein (20%), fiber (5%), fat (3.5%), ash (6.5%) as well as vitamin mixture. Each 100 g of the standard protein diet which is 20% casein contains casein (20 g), sucrose (70 g), salt mixture (4 g), oil and oil-soluble vitamins (5 g), and (0.6 g) vitamin mixture in starch [42]. To induce malnutrition, the animals were fed a low protein (PM) diet (10% casein diet was chosen for this study) with the same composition as the standard protein diet. At the beginning of the experimental period, animals were divided into two sets either fed NF or PM to induce malnutrition for three weeks. At the end of this period, blood samples were withdrawn from the retro-orbital venous plexus of pups from both sets (*n* = 10) to ensure their nutrition status by determination of serum albumin levels. Blood samples were collected in pre-cooled EDTA tubes and kept on crushed ice until centrifugation (10 min at 3000 rpm at 4 °C) to obtain serum samples. 

Subsequently, MSG (0.4 g/kg) [40,43] and MH (60 mg/kg) [41] were incorporated in the diet with either NF or PM groups according to the experimental design and administered ad libitum over a period of eight weeks.

### 4.4. Experimental Design 

**Figure 14:**
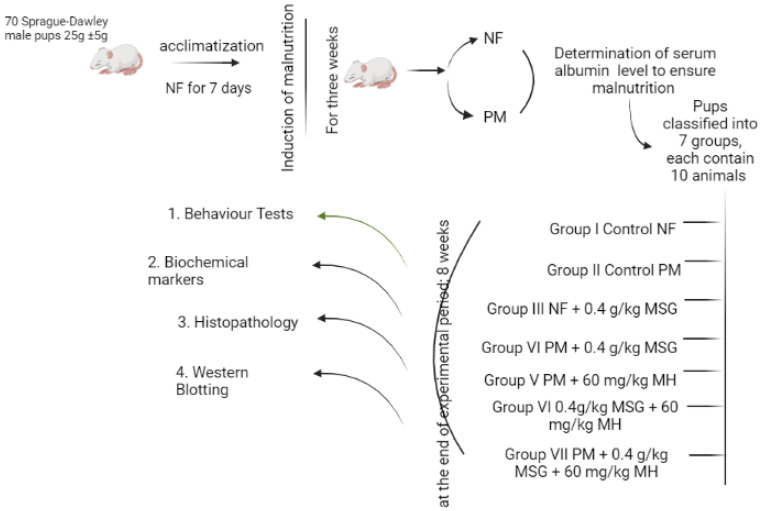
Investigating the protective mechanism of MH against MSG-induced malnutrition in NF and/PM rat pups.

Rat pups were randomly divided into seven groups (Figure 14) of 10 rats each. Group I, III, and VI were NF fed, and group II, IV, V, and VII were PM fed. Group I rats served as normal control NF and was administered daily saline only. Group II served as PM control animals. Group III received NF + 0.4 g/kg MSG, group IV received PM + 0.4 g/kg MSG and group V received PM + 60 mg/kg MH. The last two groups VI and VII received NF + 0.4 kg/g MSG + 60 mg/kg MH and PM + 0.4 kg/kg MSG + 60 mg/kg MH, respectively. At the end of the experiment, animals were exposed to Y-maze task, open field and swimming tests as described in [44]. After 24 h from the behavioral tests, all animals were anesthetized by ketamine (80 mg/kg, i.p.) then euthanized by cervical dislocation, and brains were immediately excised and rinsed with ice-cold saline. From each animal group (n = 10), the brain of four pups was fixed in 10% neutral buffered formalin for 48 h for histopathological evaluation. Collected brains of the other six rats were divided into two hemispheres; one hemisphere was homogenized in 0.1 M phosphate buffer (pH 7.4) as 10% homogenate of brain tissue. The homogenates were centrifuged at 4000 rpm for 15 min at room temperature to give 10% brain supernatants that were used for estimation of biochemical markers. The other remaining hemispheres were frozen at −80 °C for Western blotting analysis.

### 4.5. Determination of Serum Albumin Content 

Serum albumin content was assessed by using commercially available ready-made kit obtained from Stanbio Laboratory Inc. (San Antonio, TX, USA) following the manufacturer’s instructions. 

### 4.6. Behavior Assessment 

#### 4.6.1. Y-Maze Task (for Assessment of Spatial Working Memory)

Short-term memory was assessed by spontaneous alternation behavior in the Y-maze task. The Y-maze used in the present study consisted of three arms (35 cm long, 25 cm high, and 10 cm wide) and an equilateral triangular central area. Animals were placed at the end of one arm and allowed to move freely through the maze for 8 min. An arm entry was counted when the hind paws of the rat were completely within the arm. Spontaneous alternation behavior was defined as entry into all three arms on consecutive choices. The percent of spontaneous alternation was calculated as (actual alternations/maximum alternations-2) × 100 [44].

#### 4.6.2. Swimming Test (for Assessment of Motor Coordination) Reflecting the Cognitive Function)

The test measures the time in seconds taken by the animal to reach the ramp (swimming time) and the swimming direction score as described [45]. Briefly, the glass tank (91.4 cm × 91.4 cm × 30.5 cm) was filled to its half with warm water (26–27 °C) and the ramp was placed in the middle of one side of the tank (endpoint). The animals were placed in the middle of the opposite side (swimming starting point) and monitored for a maximum of 3 min during which the animal should reach the ramp. The behavior of rats in the swimming apparatus was evaluated by the following parameters: (A) the swimming time till reach the ramp with forepaws and (B) the direction score was evaluated as follows; score 4: the rat swims directly to the ramp; score 3: the animal reaches the ramp through either right or left direction; score 2: the animal reaches the ramp after swimming in both directions; score 1: the animal swims randomly then reach the ramp during 3 min and score 0: the rat swims in all directions, floats passively in the water and cannot reach the ramp within the maximal.

#### 4.6.3. Open-Field Test (OFT)

OFT was used to measure the locomotor, latency, rearing, and grooming activity of rats in an open-field paradigm. The apparatus consists of a rectangular box (40 × 50 × 63 cm) with a floor divided into 20 (10 × 10 cm) rectangular units. The animals were gently placed in the right corner of the open-field and allowed to freely explore the area for 5 min. Four parameters were quantified throughout this test: the ambulation frequency (the total number of squares that the animal crossed with all 4 limbs), latency time (time taken by the animal to start moving), rearing frequency (number of times the animals stood on their hind paws) and grooming frequency (number of times the animals washing or mouthing of forelimbs, hind-paws, face, body, or genitals). The open-field was cleaned with a 5% water-ethanol solution before behavioral testing to eliminate possible bias due to odors left by previous rats and as previously reported by [46].

### 4.7. Enzyme-Linked Immunosorbent Assays (ELISA)

Using rat-specific enzyme-linked immunosorbent assay (ELISA) commercially available kits following the manufacturer’s instructions, the following parameters were determined in 10% brain supernatant; Serotonin (5-HT) (Cat.# E-EL-0033, Elabscience, Wuhan, China), tumor necrosis factor-alpha (TNF-α) (Cat.# ab181421, Abcam, UK), apoptosis inducing factor (AIF) (Catalog# CSB-EQ027577HU, Cosmo Bio USA, Carlsbad, CA, USA), Glial fibrillary acidic protein (GFAP) (Cat.# NS830, Sigma-Aldrich, Burlington, MA, USA), interleukin-1β (IL-1β) (Cat.# 423501, BioLegend, San Diego, CA, USA) and brain derived neurotrophic factor (BDNF) (Cat.# BEK-2211-CE, Biosensis, USA). Noradrenaline (NA) (Cat.# CSB-E07870m), Dopamine (Cat.# CSB-EQ027496FI) and Glutamate (CAT. No. CSB-EQ027987HU) were purchased from Cosmo Bio, USA. 

### 4.8. Colorimetrical Analysis

Calcium, malondialdehyde (MDA), superoxide dismutase (SOD), and total antioxidant capacity (TAC) were calorimetrically assessed in 10% brain supernatant by commercially available kits supplied by Bio diagnostic, Inc., Giza, Egypt. Brain lipid peroxidation was measured relevant to MDA level using thiobarbituric acid. SOD enzyme activity was determined based on the ability of the enzyme to inhibit the phenazine methosulphate-mediated reduction of nitroblue tetrazolium dye. Finally, the antioxidant reaction with exogenously provided H_2_O_2_ was used for TAC assessment [47,48].

### 4.9. Western Blotting Analysis

Western blotting was used in the assessment of nuclear factor erythroid 2-related factor 2 (Nrf2), heme oxygenase-1 (HO-1), nuclear factor kapa B (NFκB), Toll-like receptor-4 (TLR-4), NOD-like receptor protein-3 (NLRP3), and caspase-1 in the brain supernatant. The Ready Prep^TM^ protein extraction kit for total protein assay provided by (Cat.# 163–2086, Bio-Rad Inc., Hercules, CA, USA) was added to each sample of the homogenized tissues according to manufacturer’s instructions. Bradford Protein Assay Kit (Cat.# SK3041, Bio basic Inc, Markham, ON L3R 8 T4, Canada) was used for quantitative of protein concentration in each sample. A 20 μg protein concentration of each sample was then loaded with an Laemmli’s buffer and then boiled at 95 °C for 5 min. Equal amounts of proteins were separated by Sodium Dodecyl Sulfate Polyacrylamide (SDS-PAGE) gel electrophoresis. The resolved proteins were transferred to nitrocellulose membranes to be probed with the respected primary antibodies. Polyclonal antibody of NF-κB was obtained from My BioSource, Inc. (Cat. # MBS631230, San Diego, CA, USA, dil. factor 1:300), caspase-1 was obtained from Thermofisher Scientific (Waltham, MA, USA, Cat. # MA5-16215, dil. factor 1:400), polyclonal antibodies against β-actin (dil. 1:1000). Visualization of the protein-antibody complex is performed by secondary horseradish peroxidase-conjugated antibody (Goat anti-rabbit IgG-HRP, Novus Biologicals, Centennial, CO, USA) with a dilution of 1: 5000. The developed signal by chemiluminescence was recorded by CCD camera-based imager. Image analysis software was used to quantify the band intensity of the target proteins against β-actin by protein normalization on the ChemiDoc MP imager (Bio-Rad, Hercules, CA, USA).

### 4.10. Gene Expression Measurement of Bcl-2 and Bax in Brain Tissue by Quantitative Real-Time PCR (qPCR) Analysis

Assessment of mRNA levels of *Bcl-2/Bax* and the housekeeping gene (β-actin) in brain tissue was performed using qPCR using Applied Biosystems step one plus equipment [49]. 

Total RNA was isolated using Qiagen tissue extraction kit (Qiagen, USA) according to the standard protocol. Reverse transcription of the extracted mRNA was performed by a sense fast cDNA synthesis kit (Cat. # BIO-65053) [50]. Data analyses were performed using an Applied Biosystem with a software version 3.1 (StepOne™, Carlsbad, CA, USA). Primer sequences shown in Table 2 were designed as previously described [51] for *Bcl-2*/Bax and [49] for β-actin.

### 4.11. Histopathological Analysis 

After 48 h after dissection, animal brains were removed from formalin solution and dehydrated by serial descending alcohol dilution. The specimens were cleared in xylene and embedded in paraffin at 56 °C in a hot air oven for 24 h. Different parts of brain paraffin blocks were sliced into 4-μm-thick sections. Tissue slides were deparaffinized in xylene and hydrated by ethyl alcohol in ascending concentrations to be stained using hematoxylin and eosin (H & E) stains [52].

### 4.12. Statistical Analysis 

Data were initially tested for normality using Shapiro–Wilk’s test, with data being normally distributed if *p* > 0.050. Data are expressed as mean ± S.E.M. and multiple comparisons were performed using one-way ANOVA followed by Tukey Kramer as a post hoc test. All statistical analysis and graphs were performed using GraphPad Prism software Ver. 5 (ISI^®^, San Diego, CA, USA). The level of significance was considered at *p* ˂ 0.05.

## 5. Conclusions

Our findings show that the chronic intake of MSG in early life has potential deleterious effects on CNS. It also shows that brain injury caused by MSG was way worse compared to those caused by malnutrition. However, the addition of MSG to malnourished pups showed worst behavior presented in significant disturbance in histopathological and biochemical parameters. MH administration exerted neuroprotective effect against memory impairment and improved locomotor activity in rodents via inhibition of oxidative stress markers/release of proinflammatory cytokines and suppression of protein expression of NF-kB/TLR4/NLRP3. These findings offer a novel view for exploiting MH as an alternative neuroprotective agent against malnutrition-induced behavior dysfunction such as ADHD.

## Figures and Tables

**Table 1:** Correlation of CD95 sensitivity with the intraexperimental quantification of the *Bax*/*Bcl-2* ratio.

Treatment Groups	*Bax* (Arbitrary Units)	*Bcl-2* (Arbitrary Units)	*Bax/Bcl-2* ratio
**NF**	1.383	1.074	1.28
**PM**	2.250	0.827	2.72
**MSG**	4.367	0.181	24.12
**PM + MSG**	7.483	0.036	207.86
**PM + MH**	1.433	1.003	1.43
**MSG + MH**	2.850	0.841	3.39
**PM + MSG + MH**	4.550	0.588	8.42

**Table 2:** List of primer sequence sets of *Bcl-2*, *Bax* and β-actin used for qPCR analysis in rat tissues.

Gene	Forward and Backward Primer Sequence
***Bax***	Forward: 5′-ATGTTTTCTGACGGCAACTTC-3′ Reverse: 5′-AGTCCAATGTCCAGCCCAT-3′
***Bcl-2***	Forward: 5′-CTACGAGTGGGATGCTGGAG-3′ Reverse: 5′- TTCTTCACGATGGTGAGCG-3′
**β-actin**	Forward: 5′-GGTCGGTGTGAACGGATTTGG-3′ Reverse: 5′-ATGTAGGCCATGAGGTCCACC-3′

## Data Availability

Data is contained within the article.

## Abbreviations

PM	Low protein diet
NF	Normal protein diet
ADHD	Attention deficit hyperactivity disorder
MSG	Monosodium glutamate
Nrf2	nuclear factor erythroid-2 related factor
HO-1	heme oxygenase-1
ROS	Reactive oxygen species
MH	Morin hydrate
ELISA	Enzyme-Linked Immunosorbent Assays
GFAP	Glial fibrillary acidic protein
AIF	Apoptosis inducing factor
H&E	Hematoxylin and eosin
OFT	Open field test
5-HT	Serotonin
TNF-α	Tumor necrosis factor-α
IL-1β	Interleukin-1 β
NFκB	nuclear factor kapa B
TLR-4	Toll-like receptor-4
GSH	Reduced glutathione
TAC	Total antioxidant count
*Bcl-2*	B-cell lymphoma-2
*Bax*	Bcl-2 associated X
BDNF	Brain-derived neurotrophic factor
NA	Noradrenaline
MDA	Malondialdehyde
Nlrp-3	NOD-like receptor protein-3
